# Are artificial intelligence large language models a reliable tool for difficult differential diagnosis? An a posteriori analysis of a peculiar case of necrotizing otitis externa

**DOI:** 10.1002/ccr3.7933

**Published:** 2023-09-19

**Authors:** Giorgia Pugliese, Alberto Maccari, Elena Felisati, Giovanni Felisati, Leonardo Giudici, Chiara Rapolla, Antonia Pisani, Alberto Maria Saibene

**Affiliations:** ^1^ Otolaryngology Unit Santi Paolo e Carlo Hospital Milan Italy; ^2^ Department of Health Sciences Università degli Studi di Milano Milan Italy

**Keywords:** computer‐assisted diagnosis, differential diagnosis, ear, infection, radiology

## Abstract

**Key Clinical Message:**

Large language models have made artificial intelligence readily available to the general public and potentially have a role in healthcare; however, their use in difficult differential diagnosis is still limited, as demonstrated by a case of necrotizing otitis externa.

**Abstract:**

This case report presents a peculiar case of necrotizing otitis externa (NOE) with skull base involvement which proved diagnostically challenging. The initial patient presentation and the imaging performed on the 78‐year‐old patient suggested a neoplastic rhinopharyngeal lesion and only after several unsuccessful biopsies the patient was transferred to our unit. Upon re‐evaluation of the clinical picture, a clinical hypothesis of NOE with skull base erosion was made and confirmed by identifying *Pseudomonas aeruginosa* in biopsy specimens of skull base bone and external auditory canal skin. Upon diagnosis confirmation, the patient was treated with culture‐oriented long‐term antibiotics with complete resolution of the disease. Given the complex clinical presentation, we chose to submit a posteriori this NOE case to two large language models (LLM) to test their ability to handle difficult differential diagnoses. LLMs are easily approachable artificial intelligence tools that enable human‐like interaction with the user relying upon large information databases for analyzing queries. The LLMs of choice were ChatGPT‐3 and ChatGPT‐4 and they were requested to analyze the case being provided with only objective clinical and imaging data.

## Graphical abstract

This computed tomography scan shows bilateral partial erosion of the bony boundaries of the middle skull base (simple arrows) with moderate soft tissue contrast enhancement (double arrows) in a case of necrotizing otitis externa case.

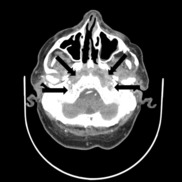

## 1 INTRODUCTION

Necrotizing otitis externa (NOE), also known as malignant otitis externa, is a potentially life‐threatening condition that primarily affects the elderly and immunocompromised individuals. It is a severe infection of the external ear canal that can progress to involve the temporal bone and adjacent structures. The diagnosis of NOE presents significant challenges due to its insidious onset, nonspecific initial symptoms, and the potential for serious complications, which can include cranial nerve palsies and intracranial involvement.^1^

Patients with NOE typically present with persistent ear pain, often out of proportion to the physical examination findings, and may also have otorrhea or hearing loss. However, these symptoms are also common in less severe conditions, which can lead to misdiagnosis and delayed treatment. Diagnostic certainty often requires a combination of clinical findings, radiological imaging, and microbiological testing. *P. aeruginosa* is the most common causative organism, found in over 90% of cases. Imaging, such as computed tomography (CT) or magnetic resonance imaging (MRI), can reveal bone erosion and soft tissue involvement, further aiding in diagnosis.^2^

The treatment of NOE is also challenging. It requires a prolonged course of antibiotics, often up to 6 weeks or longer, and the selection of the appropriate antibiotic requires antibiotic susceptibility testing due to the risk of antibiotic‐resistant *Pseudomonas*. Surgical debridement may be required in severe cases or when there is a failure to respond to medical therapy. Overall, the management of NOE requires an aggressive and multidisciplinary approach, and even with optimal treatment, the condition carries a significant risk of morbidity and mortality.^3^

NOE represents therefore an underappreciated and complex disease, both from a diagnostic and from a therapeutic perspective. It potentially requires exploring several differential diagnoses and therefore represents an optimal benchmark for testing the capabilities offered in terms of clinical management by artificial intelligence (AI) tools. Potential medical applications of such tools, to assist either in diagnosis, therapeutic decisions, and prediction of outcomes, have attracted considerable interest since its early foundations in the middle of the last century.^4^ To date, the majority of AI studies in medicine focus on its applications in radiology, including in the head and neck region.^5^, ^6^

Chat Generative Pre‐Trained Transformer (ChatGPT), an example of a large language model (LLM), is an easily accessible and user‐friendly AI tool that has gained recent attention due to its ability to textually interact with near‐human capability, due to the tool's extensive training on a wide range of texts available on the internet. Despite its apparent ease of use and unlimited capabilities, this tool application has been only minimally explored, particularly in more niche settings such as otolaryngology.^7^

The aim of this case report was therefore to employ this complex case of NOE to evaluate and present the capabilities of the aforementioned LLM in analyzing clinical data in an extremely niche setting. We submitted the case a posteriori to ChatGPT‐3 and ChatGPT‐4 in the form of objective‐only data (clinical history and imaging reports) and prompted the AI to analyze the data and explore potential differential diagnoses, comparing its answers with the real‐life clinical scenario.

## 2 CASE PRESENTATION

A 78‐year‐old man was referred by another otolaryngology service to our inpatient clinic. The patient reported left‐side otalgia and ipsilateral hearing loss beginning 2 months prior, following initial otorrhea, and showed progressive worsening.

The patient's medical records reported hypertension, dyslipidemia, Stage III chronic renal failure, and recently diagnosed Type II diabetes. Three years earlier, the patient had also undergone functional endoscopic sinonasal surgery for chronic rhinosinusitis and reported no known otological infections.

At the time of admission, his daily therapy was: subcutaneous insulin (lispro insulin TID [6, 10, and 10 units] and glargine insulin QD [10 units]); oral polyunsaturated fatty acids supplements 1 g QD; oral esomeprazole 20 mg QD; oral simvastatin 10 mg QD; oral sodium alginate/sodium bicarbonate gel 500 BID; oral folic acid supplement 5 mg QD; oral amlodipin 10 mg QD; oral calcium carbonate 500 mg BID; oral dexamethasone 20 mg QD; and oral doxazosine 2 mg, QD.

Two months before arriving at our clinic, he experienced the onset of the reported symptoms and underwent nasal endoscopy, which showed a hyperemic lesion almost totally obstructing the nasopharynx and bilaterally extending to the tubal ostia. Given this finding, the patient subsequently underwent MRI of the head with a report of “a wide, bilateral median‐paramedian lesion of the nasopharynx, which obstructed the posterolateral pharyngeal recesses and the auditory tubes and invaded the surrounding tissues; with an almost complete occupation of the left parapharyngeal space, including the neurovascular structures, which still appeared recognizable, along with infiltration and erosion of the left side of the clivus, and possible involvement of the hypoglossal canal. The lesion showed intense enhancement after administration of contrast medium.”

**FIGURE 1:**
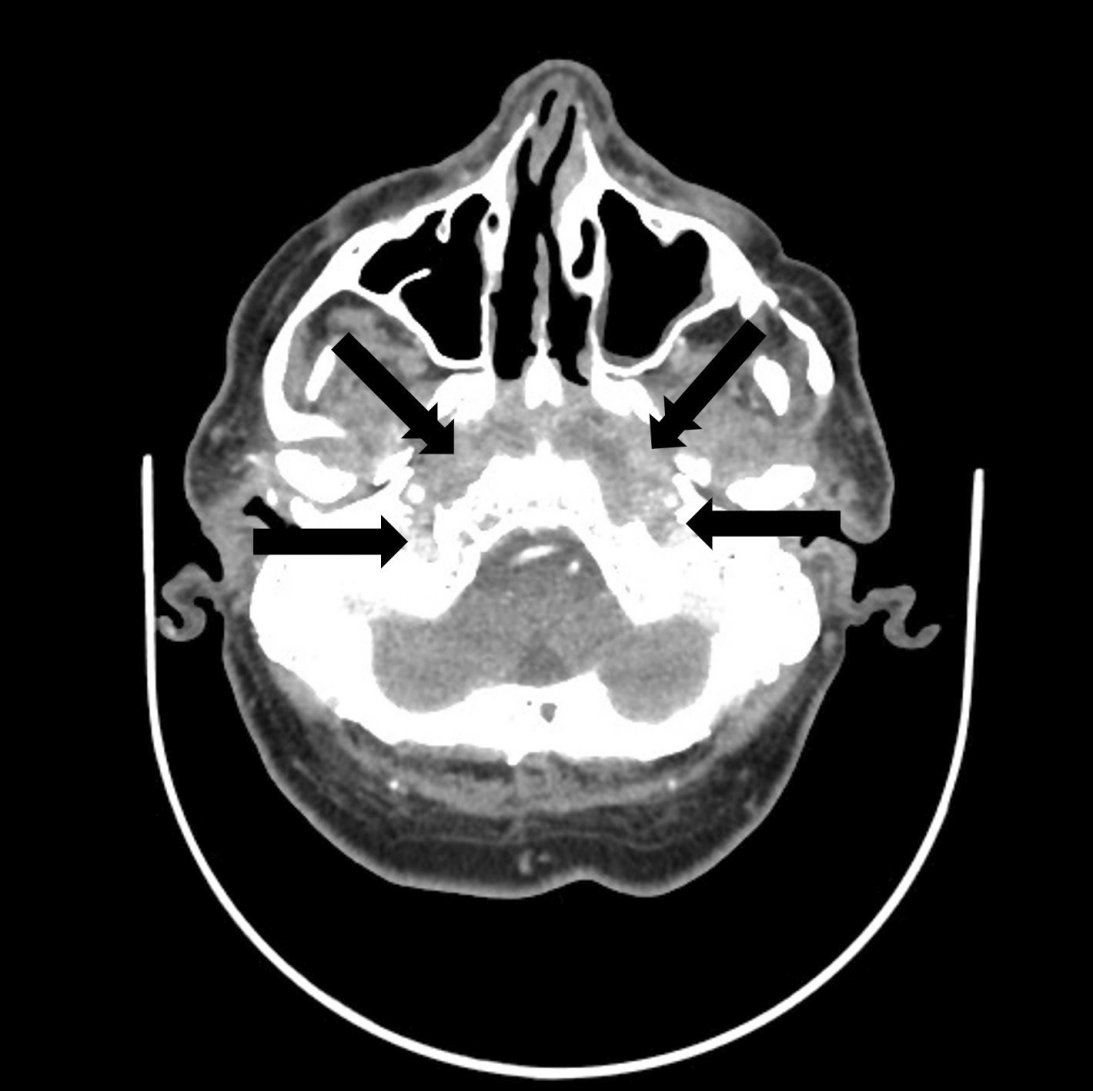
Axial CT scan showing bilateral partial erosion of the bony boundaries of the middle skull base (simple arrows) with moderate soft tissue contrast enhancement (double arrows).

**FIGURE 2:**
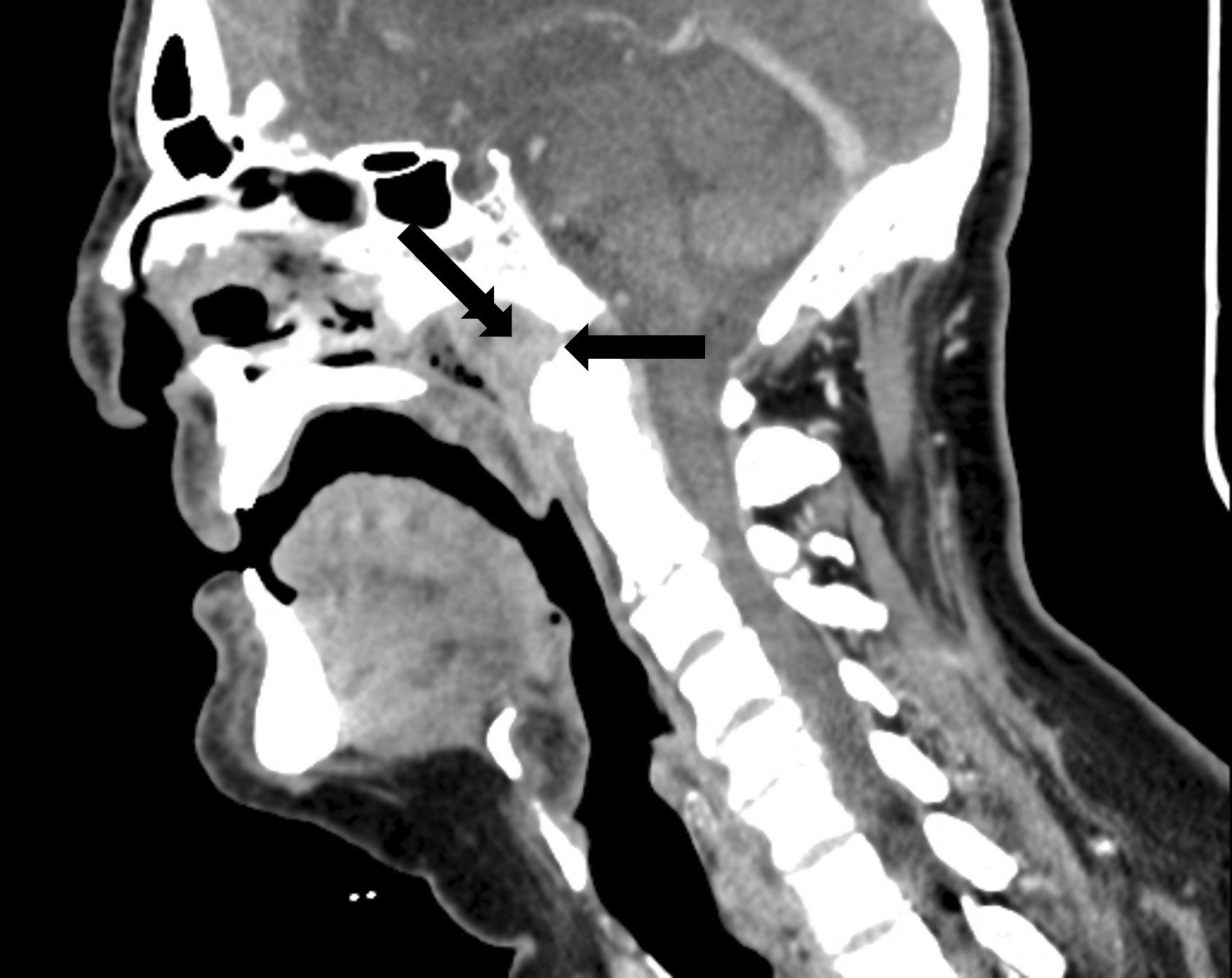
Sagittal CT scan showing partial clival erosion (simple arrow) with moderate soft tissue contrast enhancement (double arrow).

**FIGURE 3:**
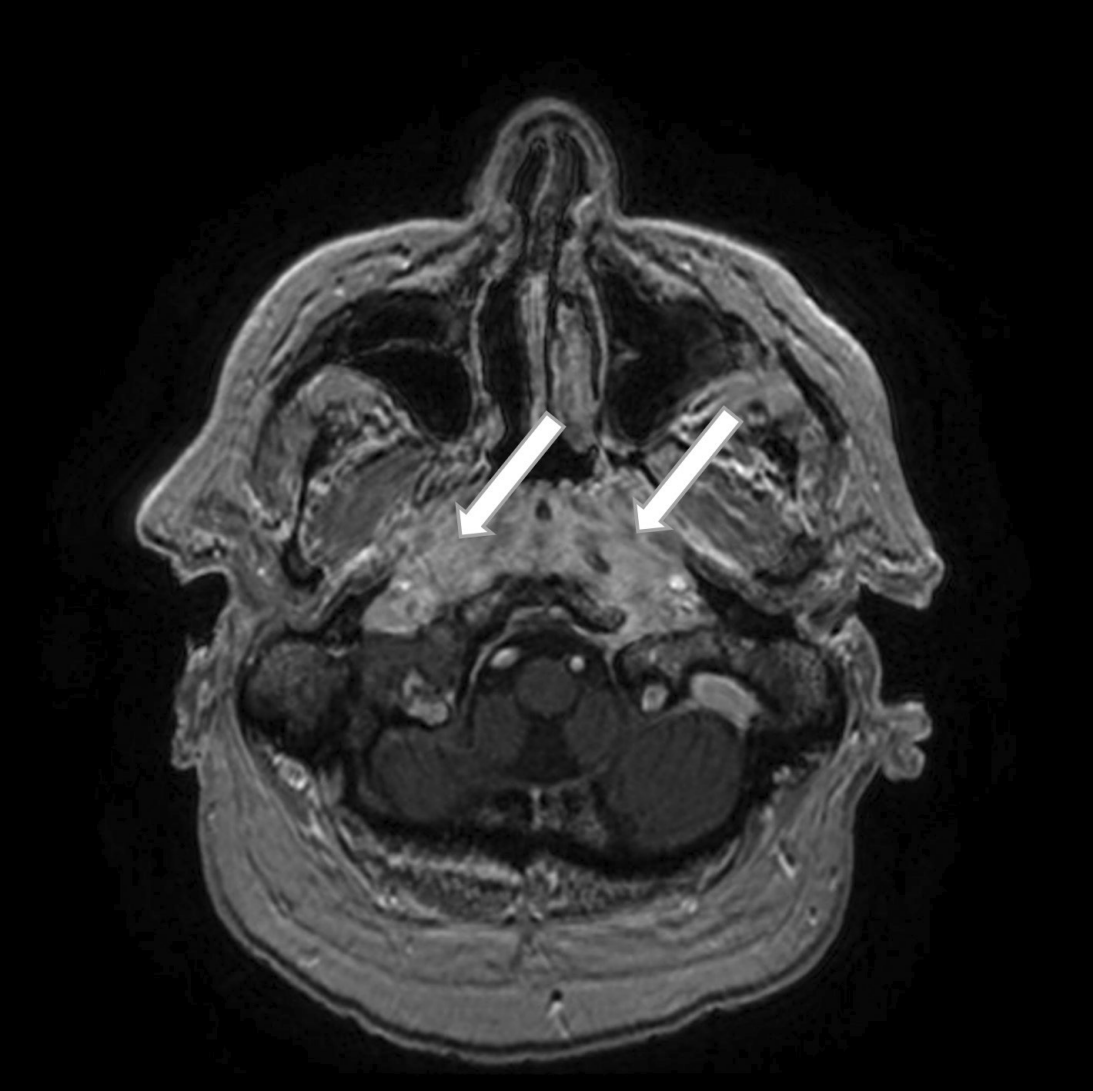
Axial fat‐sat MRI scan showing a bilateral contrast‐enhanced lesion in the rhinopharynx (simple arrows).

**FIGURE 4:**
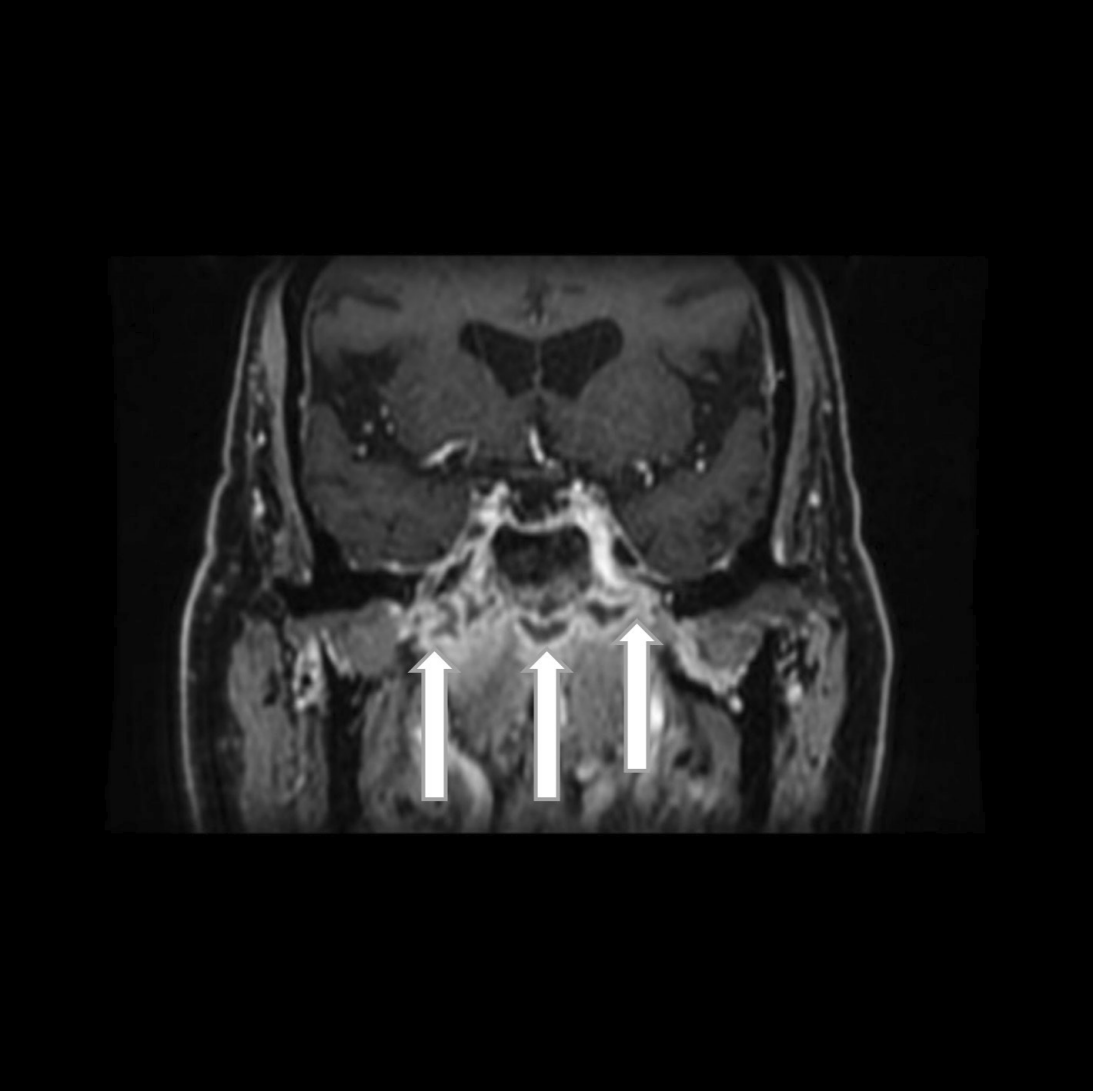
Coronal T1‐weighted MRI scan showing bilateral contrast‐enhancement along the middle skull base with some degree of necrosis (simple arrows).

**FIGURE 5:**
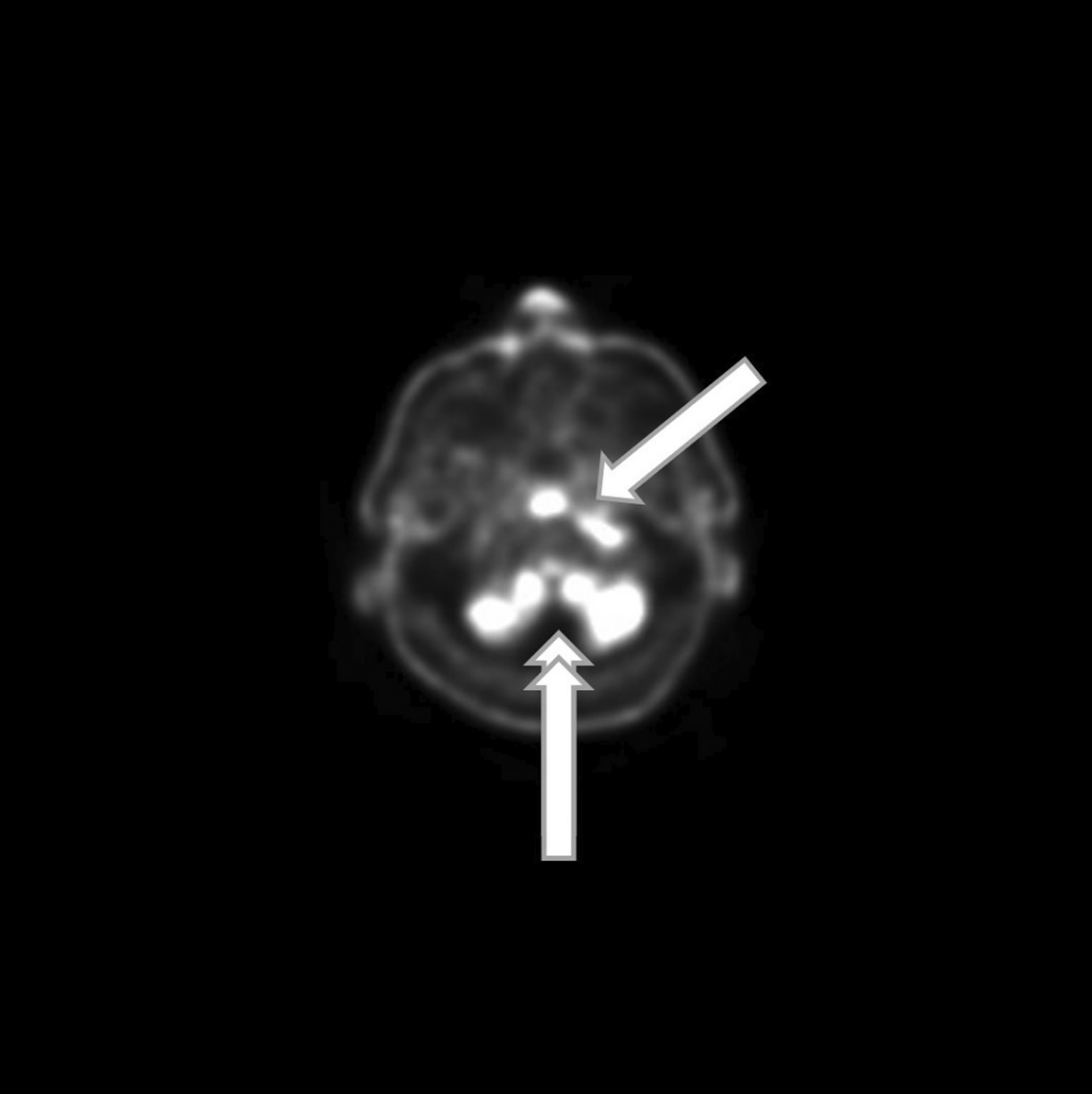
PET scan showing 18‐fluorodeoxyglucose uptake along the middle skull base and extending toward the left side (simple arrow). Note that posteriorly the cerebellum is highlighted by a normal 18‐fluorodeoxyglucose uptake (double arrows).

The inconsistent symptoms, only partially related to the otogenic origin of the disease, and confounding imaging, with bony skull base erosion and rhinopharynx contrast medium uptake, led to the hypothesis of a neoplastic disease. Based on this suspicion of a neoplastic lesion, the patient was admitted to the Oncology Unit in the hospital. After admission, he underwent further radiological imaging (i.e., total body CT, another head MRI, and total body positron emission tomography (PET); see Figures 1, 2, 3, 4, 5), with radiology reports reinforcing the neoplastic hypothesis.

To confirm this speculative diagnosis, the patient underwent a biopsy of the lesion three different times, and all biopsies yielded negative results upon histopathological examination.

Due to the difficulty in obtaining a diagnostic specimen of the lesion with endoscopy, the patient was evaluated by a neurosurgeon and subsequently referred to our clinic for a neuronavigation‐guided biopsy under general anesthesia.

Upon endoscopic evaluation, the rhinopharynx appeared free of disease, and the mucosa appeared intact, with no evidence of any anomaly that could be interpreted as a neoplasm.

Based on this finding and the medical history of the patient, our first diagnostic proposal was osteomyelitis of the skull base resulting from NOE. We still wanted to rule out any potential malignancy, however, and obtained the appropriate specimens for histology and cultures. We performed another MRI for the neuronavigation protocol and noted a dimensional increase of the lesion, with bilateral involvement of the neurovascular structures and invasion of the left mixed nerve canal. A few days after this second MRI, the patient reported dyspnea and dysphagia. Endoscopy indicated left hemi palate and laryngeal mild palsy with normal airway patency. Given the rapid evolution of the patient's symptoms, he was admitted to our ward, and a biopsy was planned for the following day. Upon admission, blood panel values were: white blood cell count (WBC) 9290 units/μl; neutrophils 7690 units/μl; C‐reactive protein (CRP) 6.9 mg/dL; erythrocytes 4.29 million/μL; hemoglobin 13.5 g/dL; mean cell volume 92.3 fL; mean cell hemoglobin 31.5 pg; mean cell hemoglobin concentration 34.1 g/dL; platelets 172,000/μL; non‐fasting glucose 149 mg/dL; creatinine 1.2 mg/dL; total bilirubin 0.7 mg/dL; alanine aminotransferase 38 units/L; aspartate aminotransferase 30 units/L; creatine kinase 40 units/L; sodium 138 mEq/L; potassium 4 mEq/L).

During surgery, we observed abundant purulent secretions at the level of the right external auditory canal, which had an intact tympanic membrane. As planned, a myringotomy was performed, enabling the suctioning of abundant glue‐like mucus secretions from the middle ear, followed by the placement of transtympanic drainage. In addition, a skin sample from the external auditory canal was harvested and sent for histology and cultures.

The nasopharynx showed highly vascularized, lymphoid‐type hyperplastic tissue. We collected some tissue samples, which revealed only inflammatory cells and no evidence of heteroplasia. Afterward, we explored all the regions involved in the lesion and took further specimens of the nasopharynx and the clivus. All tissue samples collected were sent for histological examination and culturing.

Based on the strong evidence of an infectious process, we set up an empirical antibiotic therapy with iv vancomycin 1500 mg QD and iv meropenem 2 g BID. When the cultures were found to be positive for *P. aeruginosa*, the therapy was adjusted by switching to cefepime’ 2 g TID.

The postoperative MRI performed a week after surgery and following the introduction of antibiotic therapy showed a significant reduction in cranial enhancement.

A few days after surgery, the patient reported a cough, a temperature of 38.5°C, and desaturation. Examination of the blood showed an increase in WBC (7040 units/μL), neutrophils (3990 units/μL), and C‐reactive protein (CRP) (53 mg/dL). Nasal endoscopy showed drainage of purulent secretions from the surgical site and toward the larynx. After suspecting aspiration pneumonia, metronidazole 500 mg QID was added to the therapy for a total duration of 7 days. Blood cultures and bronchoalveolar lavage (BAL) yielded negative results for bacteria, viruses, or malignant cells. The latter though showed positivity for *Candida albicans*.

A chest CT scan showed mild bilateral pleural effusion and multiple areas of ground‐glass opacity, primarily in the right lung.

To rule out congestive heart failure, echocardiography was performed and showed evidence of mild pericardial effusion and mild tricuspid insufficiency, but no impairment of heart function.

Two weeks after surgery, the patient was transferred to the Infectious Disease ward to receive medical treatment. Due to the rise in blood creatinine, cefepime was switched to ceftazidime 2 g TID and continued for the remainder of the patient's hospital stay. Concurrently, due to the onset of inferior right limb edema and detection of femoropopliteal thrombosis, anticoagulant therapy with apixaban 5 mg BID was initiated. All hematological assessments were normal.

A month after surgery, the patient was considered clinically stable and was discharged from the hospital. At the time of discharge, inflammatory indexes had progressively improved (WBC 7320 units/μL, CRP 8.8 mg/dL). Oral ciprofloxacin 500 mg TID for 2 weeks, later reduced to BID for another 6 weeks, and topical dexamethasone and ciprofloxacin in the left ear duct for 8 weeks were prescribed. At the end of the antibiotic therapy, the patient was in overall good condition, with no unusual symptoms or complaints, and blood inflammatory indices had returned to the normal range. Nasal endoscopy showed complete healing of the sphenoidotomy, with no indication of any purulent content, and complete resolution of the laryngeal and palatal palsy. Otoscopy revealed left‐side tympanic perforation without evidence of infection. On the right side, transtympanic drainage was patent and correctly placed. The patient was scheduled to partake in regular otolaryngological follow‐up every 2 weeks, while maintaining daily borate ear washes.

**AI analysis** Prior to hospital admission, we submitted the patient's information to ChatGPT‐3 and ChatGPT‐4 bots. We presented the case based only on the objective clinical symptoms, provided temporal indications of their presentation, and reported all diagnostic examinations the patient had undergone. In addition, we described the radiological exams and reported only the objective data, without mentioning any interpretation by the radiologist.

The prompt we used for the bot was as follows:

Pretend you are an otolaryngologist in an inpatient clinic, and you are seeing this patient for the first time. A 78 years old man comes referring left side otalgia and hearing loss. These symptoms started two months ago, after an episode of otorrhea, ang progressively got worse.

In his medical history: hypertension, dyslipidemia, chronic renal failure (III stage), a recent diagnosis of type II diabetes. He underwent sinonasal surgery three years ago. He has no history of ears' affections. Two months ago (February 2023), at the beginning of the reported left ear's symptoms, he underwent a nasal endoscopy which showed a hyperemic neoplasm almost totally obstructing the nasopharynx and reaching the tubal ostia bilaterally. Given this finding, soon he underwent a Maxillofacial MRI […].

Therefore, the patient has been hospitalized in the Oncology Unit, where he underwent further radiological exams. […].

The ChatGPT‐3 response was as follows:

Based on the patient's symptoms and medical history, as well as the results of the various imaging tests, some of the possible differential diagnoses that might be considered include:

1. *Nasopharyngeal carcinoma*: This is a rare type of cancer that arises in the nasopharynx and can cause ear pain, hearing loss, and other symptoms. The imaging findings described in the case are highly suggestive of nasopharyngeal carcinoma.
2. *Squamous cell carcinoma*: This is a type of cancer that can occur in the mucous membranes of the head and neck, including the nasopharynx. It can also cause similar symptoms to those described by the patient.
3. *Lymphoma*: This is a type of cancer that affects the lymphatic system and can cause lymph node swelling and other symptoms. It is less likely to be the cause of the patient's symptoms given the imaging findings.
4. *Infection*: An infection of the ear or nasopharynx can also cause ear pain and hearing loss, but the imaging findings are not consistent with an infectious process.
5. *Benign neoplasms*: Although less likely given the imaging findings, benign tumors such as schwannomas or meningiomas can sometimes cause similar symptoms.

The CHATGPT4 response was as follows:

I'm not a doctor, but I can try to help you think through this case. Based on the patient's symptoms and the imaging findings, some possible differential diagnoses to consider could include:

1. *Nasopharyngeal carcinoma (NPC)*: The patient's age, the presence of a nasopharyngeal mass, and the imaging findings indicating invasion of adjacent structures and cranial nerves are suggestive of NPC.
2. *Lymphoma*: The involvement of lymph nodes and the mass in the nasopharynx could also raise the possibility of lymphoma.
3. *Other head and neck malignancies*: Other malignancies, such as squamous cell carcinoma or adenocarcinoma, could be considered based on the patient's age and the imaging findings.
4. *Parapharyngeal space tumor*: Although less likely given the extent of involvement, a tumor originating from the parapharyngeal space could be considered.
5. *Inflammatory/infectious processes*: Although less likely given the imaging findings, it is important to consider inflammatory or infectious processes that could cause similar symptoms, such as fungal infections, granulomatous diseases, or autoimmune conditions.

## 3 DISCUSSION

Based on developments achieved to date, AI has shown great effectiveness in making diagnoses from imaging analysis,^8^ although less insight is available on the ability of AI to interpret more complex, text‐based depictions of clinical scenarios. Regarding the head and neck setting, in particular, AI has been found to be reliable in analyzing maxillofacial radiological imaging for dental bone pathology,^9^ in addition to the differential diagnosis of tumors of the head and neck.^10^, ^11^ Moreover, the effectiveness of AI in the diagnosis of tumors has also been demonstrated in the recognition of precancerous and cancerous lesions based on specimen images.^12^ Thus, the growing role of AI in performing repetitive analytic tasks^13^ and assisting with complex forecasts^14^, ^15^ has been confirmed. With regard to otological disturbances, AI has shown its effectiveness in making a differential diagnosis of otitis based on otoscopy imaging,^16^ and in recognizing cholesteatoma from chronic otitis media on a CT scan.^17^

Therefore, we can state with reasonable confidence that submitting visual data, or a clinical history, to AI enables an accurate assessment of the patient in near‐real time. However, it appears that the capabilities of AI in making a differential diagnosis do not exceed the physicians' expertise, and it has been shown that having an AI‐driven list of differential diagnoses does not meaningfully affect a physician's accuracy in making a diagnosis.^18^ Moreover, LLM represents an additional, user‐friendlier tool that is readily available to any clinician and has revolutionized the perception of AI by the general public and specialists alike, with the latter recognizing the role of AI in providing simple and understandable information.^7^

What is still not clear, however, and what we aimed to understand with this preliminary evaluation, is whether LLM can provide distinctive and useful insight into difficult clinical cases, by decontextualizing information and removing the physician's biases from the clinical evaluation.^19^ Even more so, we are still uncertain about LLM's role in decision‐making in the context of niche applications and with negligible internet presence, such as NOE. This latter application is of utmost interest, not only from a medical point of view, but accurate assistance with difficult scenarios would make these tools extremely valuable, including from an information technology standpoint, as LLM tends to produce more accurate replies when the data pool they are trained in is large.

As such, we chose to submit this NOE case to an LLM chatbot to see whether it could more easily make a diagnosis of a case that was complex and problematic for many specialists in different fields (i.e., otolaryngologists, oncologists, and neurosurgeons) to solve.

What this report illustrates is that the answers produced by both LLMs substantially retraced as a first option the same diagnostic hypothesis initially made by physicians (i.e., a neoplastic condition). Each LLM gave five differential diagnoses, and the first three were different kinds of potential malignancies. Infection was considered as an option (fourth option for ChatGPT3 and fifth option for ChatGPT4), but in both cases, the LLM stated that such a diagnosis was not consistent with available imaging data, thus somehow using the radiology report information only shallowly. Furthemore, though both LLMs hinted at a potential infectious process, neither of the two actually suggested a potential NOE, thus completely failing the primary objective of our evaluation. It must be taken nevertheless into account that being able to appreciate the real radiologic images and not only analyzing the reports represents a significant advantage for the clinician, and AIs capable of integrating both text and native graphic data are not yet available to the general public. Furthermore training AIs on the interpretation of radiologic images might represent still a challenge both from an information technology, time‐expenditure, and sensible data management perspective. Another apparent limitation of LLMs in this context is that they are not able to integrate the imaging data and follow the presented clinical history. While in the physician's perspective repeated failure to obtain a positive malignancy specimen and absence of disease progression over the course of several months tends to make a malignancy less plausible, LLMs seem incapable of interpreting data with consequentiality and along a timeline. Despite the vastity of their database, LLMs still lack the ability to gather inferential information from complex contexts such as this case. Therefore, we might conclude that, in its current state, AI does not appear yet as a sufficiently powerful tool for supporting differential diagnoses of complex clinical cases, in which a physician's clinical experience remains essential for reaching the best solution. It appears that AI, in its current iteration, has the tendency to behave like an inexperienced clinician by overlooking potential differential nuances and going straight for the most obvious response, as if it were a modern version of Occam's razor.

Understandably enough, given the recent developments in the field, the present case can be considered a preliminary application of LLM in NOE. Nevertheless, NOE represents a well‐described clinical scenario, and its complex management is well‐described by this difficult differential diagnosis scenario. On the other hand, as stated above, NOE is a rather obscure condition to the general public with little internet coverage (i.e., 14,400 Google search hits, against 3,240,000 Google search hits for “nasopharyngeal carcinoma”, the most probable diagnosis made by ChatGPT‐3 and 4). This observation could also explain why the answers between the two versions of the chatbot do not significantly differ. For a marginalized subject, with little internet exposure, an enlarged database is unlikely to provide any tangible progress. The only real content difference between the answers of the two chatbots was the widening of the generic “inflammatory disease” part provided by ChatGPT‐4, which was devoid of any clinical implication. This observation could imply that diseases more thoroughly covered by internet sites may be more easily targeted by LLM, thereby inducing a non‐negligible bias.

In this context, it is worth being reminded that NOE is a complex infection with diagnostic and management parameters that still lack common guidelines. Identifying the clinical signs of NOE, and the ability to make a fast and correct diagnosis, is critical, as the disease is rapidly progressive and can lead to potentially fatal consequences. Moreover, treatment should always be individualized and involve multidisciplinary cooperation among specialties. In our case, the collaboration between our otolaryngology department and infectious disease specialists enabled us to tailor the antibiotic treatment to the patient's general condition, as well as to the antibiogram obtained from surgical specimens.

Typical management of NOE includes systemic antibiotics and the strict control of diabetes. Surgical therapy is not usually recommended, although it can be an option for pain relief pain in refractory cases and for evacuating NOE‐related skull base abscesses.^20^, ^21^, ^22^

## 4 CONCLUSION

In conclusion, LLMs in our study failed to prove capable of supporting complex differential diagnosis in a niche context. Despite the excellent progress that has been made with AI to date, it seems that these tools are not sufficiently reliable to employ for differential diagnoses in patients. With regard to the specific aim of our study, LLM appears to perform as an inexperienced physician, with a tendency to create medical scenarios and draw conclusions for the most obvious scenario; therefore, AI does not provide, at present, solid and reliable assistance to the work of experienced medical staff in a clinical setting. The application of physicians' expertise, along with multidisciplinary collaboration, still appears to be fundamental for the diagnosis of disease and therapeutic decision‐making in complex clinical cases. Future developments of LLM that include a wider knowledge base may, however, enable a wider role in the management of complex clinical scenarios.

## AUTHOR CONTRIBUTIONS

**Giorgia Pugliese:** Investigation; visualization; writing – original draft; writing – review and editing. **Alberto Maccari:** Conceptualization; data curation; methodology; supervision; writing – review and editing. **Elena Felisati:** Data curation; methodology; validation; writing – original draft. **Giovanni Felisati:** Conceptualization; supervision; validation; writing – review and editing. **Leonardo Giudici:** Data curation; investigation; methodology; visualization. **Chiara Rapolla:** Investigation; validation; writing – original draft. **Antonia Pisani:** Conceptualization; investigation; validation; writing – review and editing. **Alberto Maria Saibene:** Conceptualization; data curation; methodology; validation; writing – original draft; writing – review and editing.

## FUNDING INFORMATION

The authors received no financial support for the research, authorship, and/or publication of this article.

## CONFLICT OF INTEREST STATEMENT

The authors have no potential conflict of interest or financial disclosures to make.

## CONSENT

Written informed consent was obtained from the patient to publish this report in accordance with the journal's patient consent policy.

## Data Availability

All data pertaining to this case report are available from the authors upon reasonable request.
